# 18F-FDG PET and PET/CT in the Localization and Characterization of Lesions in Patients with Ovarian Cancer

**Published:** 2012-01-18

**Authors:** M.G. Caprio, D. Capacchione, C. Mainolfi, A.M. Spera, B. Salvatore, L. Cella, M. Salvatore, L. Pace

**Affiliations:** 1 Azienda Ospedaliera Universitaria Federico II, Napoli, Italia; 2 IRCCS CROB, Rionero in Vulture, Italia; 3 Dip. Scienze Biomorfologiche e Funzionali, Facoltà di Medicina e Chirurgia, Università degli Studi di Napoli Federico II, Italia; 4 I.B.B., C.N.R., Napoli, Italia; 5 Dip. di Medicina e Chirurgia, Università degli Studi di Salerno, Italia

**Keywords:** Ovarian Cancer, ^18^F-FDG, PET/CT

## Abstract

**Aim:**

The aim was to compare the imaging findings of ^18^F-fluorodeoxyglucose (^18^F-FDG) PET and integrated PET/CT in patients with primary, recurrent or metastatic ovarian cancer.

**Materials and methods.:**

21 women with ovarian cancer were evaluated. All patients had a integrated PET/CT scan. Localization, infiltration and uptake intensity of [^18^F]FDG were evaluated on PET and PET/CT. The certainty of localisation and characterisation was scored on a 3 point scale (L1 definite localisation; L2 probable localisation; L3 uncertain localisation; C1 benign; C2 equivocal; C3 malignant).

**Results.:**

PET scored as L1 54 lesions (44%), as L2 51 (42%), and as L3 17 (14%). On the other hand, PET/CT scored as L1 120 lesions (98%), as L2 2 (2%), and none as L3. Thus PET/CT allowed a better localization in 54% of lesions. Moreover, PET scored as C1 25 lesions (20%), as C2 62 (51%), and as C3 35 (29%). On the other hand, PET/CT scored as C1 57 lesions (47%), as C2 13 (11%), and as C3 52 (42%). Thus PET/CT allowed a sensible reduction in the number of equivocal lesions (40%). Even when patients were subgrouped on the basis of clinical stage of the disease, PET/CT was capable of better definition of the lesions either for localization and for characterization.

**Conclusions:**

In patients with ovarian cancer, PET/CT allows better anatomical localisation of pathologic uptake providing high accuracy for staging and restaging of ovarian cancer when compared with PET alone.

## INTRODUCTION

Ovarian cancer is the third most common of all female reproductive system cancer in terms of frequency, but it determines 50% of deaths. Diagnosis of ovarian cancer is relatively late, while the use of ultrasound examination and tumoral markers dosage (eg. CA125) promotes early detection of cancer.

Clinical stage is the most important prognostic factor in ovarian cancer. Actually, the overall five year survival rate is 80% for stage I, 50% for stage II, 30% for stage III and less than 8% for stage IV (1). As a consequence a correct staging is relevant, and imaging plays an important role with a better accuracy of Computed Tomogaphy (CT) and Magnetic Resonance (MR) in advanced stages than ultrasound (2).

Positron Emission Tomography (PET) with ^18^F-fluorodeoxyglucose (^18^F-FDG) may be used for diagnostis as well as for staging and re-staging of patients with ovarian cancers. The introduction of integrated PET/CT, allowing the visualization of either functional and morphological information on fused images, improved diagnostic significantly due to a reduction in False Positive and False Negative values.

The aim of this study is to compare results acquired with FDG PET to integrated FDG PET/CT imaging technique in patients with ovarian cancer.

## MATERIALS AND METHODS

### Population

A total of 21 women with ovarian cancer (age range 29 - 80 years, mean 53 ± 14) constituted the study group. Two of them were in staging phase, 12 in chemotherapeutical follow-up, 7 in post-surgical stage. All have been staged according to the FIGO (International Federation of Gynecology and Obstetrics) stadiation as follows: 4 were at I stage, 1 was at II stage, 5 were at III stage and 11 were at IV stage. Tumour markers such as CA125, αFP and βHCG have been dosed: marker’s values were increased in 17 patients and were normal in 5 cases. (Table I).

### Image Acquisition

All patients fasted for 8 hours before imaging. PET/CT was obtained on a commercial PET/CT scanner (Discovery LS; GE Milwaukee, WI, USA), which combines an Advance NXi PET scanner and a Light Speed Plus four row MDCT system. In all studies, PET/CT imaging was acquired 60 minutes after intravenous administration of 370 – 444 MBq of ^18^F-FDG. MDCT (pitch 1.5; 120 mAs; 120 kVp) was performed without intravenous and/or oral contrast medium as part of the PET/CT scan. PET scanning was subsequently performed with 4 minutes per bed position and six to eight bed positions per patient, depending on patient height. Raw CT data were reconstructed into transverse images with a 4.25-mm section thickness. Sagittal and coronal CT images were generated by reconstruction of the transverse data. Raw PET data were reconstructed with and without attenuation correction into transverse, sagittal, and coronal images. Attenuation correction was based on the CT attenuation coefficients, which were determined by iterative reconstruction. Blood glucose level was determined in all patients before ^18^F-FDG administration and a cut-off value of less than 140 mg/dL was considered appropriate to perform examination.

### Data analysis

Two nuclear medicine physicians, unaware of the patients’ clinical history, blindly examined PET images, evaluating localization and characterization and compared them to co-registered PET/CT images. Maximum standardized uptake values (SUVmax) were determined by using vendor-provided software (Volumetrix for PET-CT; GE Healthcare) on PET scans. Region of interest diameter was set at 1 cm. SUVmax was body weight corrected.

Anatomical localization and lesion characterization were the two parameters used for the evaluation of each lesion. For both parameters a three point score was used: L1 (definite localization), L2 (probable localization), L3 (uncertain localization); C1 (benign), C2 (equivocal), C3 (malignant) (Table II). Weighted Kappa Statistical Analysis for both PET and PET/CT to evaluate the interobserver variability in the assessment of the localization and the characterization (3).

## RESULTS

Both PET and PET/CT identified 122 lesions in 21 patients. Of the 122 lesions PET scored 54 lesions (44%) as L1, 51 lesions (42%) as L2, 17 lesions (14%) as L3. PET/CT scored 120 lesions (98%) as L1, 2 lesions (2%) as L2 and 0 lesions as L3. Table III shows the comparison between the two imaging methods. PET/CT allowed a better localization in a large number of lesions (54%).

Of the 122 lesions PET characterized 25 lesions (20 %) as C1, 62 lesions (51 %) as C2, 35 lesions (29 %) as C3. PET/CT characterized 57 lesions (47%) as C1, 13 lesions (11 %) as C2, 52 lesions (42%) as C3. Table IV shows the comparison between the two imaging methods. PET/CT allowed a sensible reduction (40%) in the number of equivocal lesions. PET/CT improves the localization of lesions in 60% of patients with stage I and II ovarian cancer and the characterization with a 43% reduction of uncertain lesions (Table V). PET/CT improves of 40% the localization and of 46% the characterization in patients with III stage ovarian cancer and improves of 60% the localization and of 32% the characterization in patients with IV stage (Tables VI and VII).

Concordance of PET for localization was 89% (109/122) k = 0.82; Concordance for characterization was 90% (110/122) k = 0.84; the level of concordance of PET/CT for localization was 100% (122/122) k = 1.0; the level of concordance for characterization was 99% (121/122) k = 0.99.

## DISCUSSION

The results of the present study show that PET/CT allows a better localization in 54% of lesions and a better characterization of tracer uptake in 40% of lesions with an higher interoperator reproducibility than PET. The role of ^18^F-FDG-PET in diagnosis and staging of primitive ovarian cancer is controversial. Older studies (4, 5) showed a sensitivity of 83–86% and a specificity of 54–86%. Rieber et al. (6) examined the role of FDG-PET in preoperative diagnosis of 103 patients with sensitivity, specificity and diagnostic accuracy values of 58%, 78% and 76% respectively. Values obtained with other methods such as MR, transvaginal sonography and histologic findings were: sensitivity 83%, 92% and 92%; specificity 84%, 59% and 84%; diagnostic accuracy 83%, 63% and 85%, respectively. More recently Fuccio et al. concluded that F-18 FDG PET/CT represents an important method in addition to other imaging modalities (transvaginal ultrasound-, and contrast-enhanced computed tomography) in the characterization of adnexal masses and in the staging of ovarian cancer patients, particularly in assessing the presence of extra-abdominal metastatic spread (7). In addition, the low value of FDG PET sensitivity is related to the high percentage of low malignity cancers and to early cancers compared to the high sensitivity of previous studies that analyzed advanced ovarian cancers.

Zinny et al. (8) studied the role of FDG-PET in the diagnosis of recurrent ovarian cancer in 106 patients under follow-up selected for secondary cytoreductive surgery and chemotherapy. Overall sensitivity of 83% and specificity of 83% were observed. Moreover, sensitivity was 94% in patients with clinical suspicion of disease, compared to 65% in patients considered clinically free. Sari et all. Showed that PET/CT is a beneficial method for detection of the recurrence, in patients with increased serum CA 125 level and negative CT findings or with normal CA 125 level and recurrence detected by CT which was performed due to clinical symptoms(9).

PET itself gives few informations on anatomic localization of lesions, making difficult to discriminate between areas of pathological uptake and physiological distribution of tracer (10, 11). The hybrid PET/CT system produces multimodal images with anatomical morphological outline useful for a better spatial localization of tracer distribution

Bristow reported a PET/CT accuracy of 81,8% in discriminating recurrent ovarian cancer (>1 cm) and a 83,3 % sensitivity (12). Sironi study analyzed the possible role of PET/CT in the evaluation of recurrent ovarian cancer and reported an high positive predictive value (89%) and a low negative predictive value (57%) (13).

Detection and exact localization of recurrent lesions are critical for guiding management and determining the proper therapeutic approach, which may prolong survival. Fluorine 18 fluorodeoxyglucose positron emission tomography (PET) combined with CT is useful for detection of recurrent or residual ovarian cancer and for monitoring response to therapy. However, PET/CT may yield false-negative results in patients with small, necrotic, mucinous, cystic, or low-grade tumors. In addition, in the posttherapy setting, inflammatory and infectious processes may lead to false-positive PET/CT results. Despite these drawbacks, PET/CT is superior to CT and MR imaging for depiction of recurrent disease. (14)

In the present study PET/CT showen a remarkably low percentage of uncertain localization (2% of lesions). In addition, characterization of lesions was improved by PET/CT. Thus, PET/CT not only allows a better localization of lesions but also plays a role in characterization. The improvement in both lesion localization and characterization was consistent in all stages of disease. These findings are in agreement with previously reported data (15). Moreover, recent studies demonstrated that FDG-PET/CT is more accurate than CT and MR in the detection of lymph node metastasis in patients with ovarian cancer (16,17).

## CONCLUSIONS

PET/CT improves the anatomical localization of lesions and the related characterization with a strong decrease of lesions considered uncertain and it shows an high reproducibility. Integrated FDG-PET/CT can be successfully used for diagnosis, staging, restaging, therapy monitoring and prognostic prediction of ovarian cancer.

## Figures and Tables

**TABLE I:** Characteristics of patients

**#**	**AGE**	**HISTOLOGY**	**STAGE**	**SURGERY**	**CHEMOTHERAPY**	**MARKERS**
1	53	endometrioid cancer	IV	yes	yes	Ca 125(a)
2	69	tubaric cancer	II A	yes	yes	Ca 125(a
3	50	serous cancer	IV	yes	yes	Ca125 (a)
4	53	clear cell cancer	IA	yes	yes	Ca125 (n)
5	72	ovarian cancer	III	yes	yes	Ca125 (n)
6	73	serous ovarian cancer	IA	yes	yes	Ca125 (a)
7	33	germ cell ovarian cancer	III	yes	yes	αFP (n)
8	50	ovarian cancer	IV	yes	yes	Ca 125(a)
9	51	ovarian cancer	IV	yes	yes	Ca125 (n)
10	51	ovarian cancer	IV	yes	yes	Ca125 (n)
11	29	choriocarcinoma	IV	yes	yes	βHCG a)
12	67	serous mucinous ovarian cancer	IA	yes	yes	Ca125 (a)
13	80	mucinous ovarian cancer	I	yes	no	Ca125 (a)
14	58	papillary serous ovarian cancer	III	yes	no	Ca125 (a)
15	74	serous cancer	IV	yes	no	Ca125 (a)
16	65	papillary serous ovarian cancer	III	yes	yes	Ca125 (a)
17	66	endometrioid cancer	IV	yes	yes	Ca125 (a)
18	50	mucinous ovarian cancer	IV	no	no	Ca125 (a)
19	43	mucinous ovarian cancer	IV	no	no	Ca125 (a)
20	72	ovarian cancer	III	yes	no	Ca125 (a)
21	62	ovarian cancer	IV	yes	no	Ca125 (a)

a: abnormal marker value

n: normal marker value

**TABLE II:** Localization and Characterization Scores

**Localization Score**	**Characterization Score**
**L1 (definite):** definite anatomical localization	**C1 (benign)** SUV ≤ 2.0
**L2 (probable):** probable localization among different contiguous anatomic sites	**C2 (equivocal)** SUV ≥ 2.0 but < 2.5
**L3 (uncertain):** localization possible only for large topographic areas	**C3 (malignant)** SUV ≥ 2.5

**TABLE III:** Localization of Lesions (N= 122): PET and PET/CT data

**localization score**	**PET**	**PET/CT**	**change PET/CT vsPET**
L1 definite	54 (44%)	120 (98%)	+ 54%
L2 probable	51 (42%9	2 (2%)	−40%
L3 uncertain	17 (14%)	0	−14%

**TABLE IV:** Characterization of Lesions (N= 122): PET and PET/CT data

**characterization score**	**PET**	**PET/CT**	**change PET/CT vsPET**
C1 benign	25 (20%)	57 (47%)	+ 27 %
C2 equivocal	62 (51%)	13 (11%)	−40 %
C3 malignant	35 (29%)	52 (42%)	+13 %

**TABLE V:** Stage I and II patients: PET and PET/CT (Patients= 5, Lesions= 30)

	**PET**	**PET/CT**	**change PET/CT vs PET**
**localization**			
L1 definite	12 (40%)	30 (100%)	+ 60 %
L2 probable	18 (60%)	0	−60%
L3 uncertain	0	0	0
**characterization**			
C1 benign	12 (40%)	24(80%)	+40%
C2 equivocal	18 (60%)	5 (17%)	− 43%
C3 malignant	0	1(3%)	+3%

**TABLE VI:** Stage III patients: PET and PET/CT (Patients= 5, Lesions= 35)

	**PET**	**PET/CT**	**change PET/CT vs PET**
**localization**			
L1 definite	19 (54%)	33 (94%)	+ 40 %
L2 probable	15 (43%)	2 (6%)	− 37%
L3 uncertain	1 (3%)	0	− 3%
**characterization**			
C1 benign	4 (11%)	14 (40%)	+ 29%
C2 equivocal	21 (60%)	5 (14%)	− 46 %
C3 malignant	10 (29%)	16 (46%9	+ 17%

**TABLE VII:** Stage IV patients: PET and PET/CT (Patients= 11, Lesions= 57)

	**PET**	**PET/CT**	**change PET/CT vs PET**
**localization score**			
L1 definite	10 (18%)	21 (37%)	+ 19%
L2 probable	23 (40%)	5 (8%)	− 32 %
L3 uncertain	24 (42%)	31 (55%)	+ 13%
**characterization score**			
C1 benign	10 (18%)	21 (37%)	+ 19%
C2 equivocal	23 (40%)	5 (8%)	− 32 %
C3 malignant	24 (42%)	31 (55%)	+ 13%
